# Correlation of p16 immunohistochemistry with clinical and epidemiological features in oropharyngeal squamous-cell carcinoma

**DOI:** 10.1371/journal.pone.0253418

**Published:** 2021-06-17

**Authors:** Chrystiano de C. Ferreira, Rozany Dufloth, Ana C. de Carvalho, Rui M. Reis, Iara Santana, Raiany S. Carvalho, Ricardo R. Gama

**Affiliations:** 1 Department of Head and Neck Surgery, Barretos Cancer Hospital, Barretos, São Paulo, Brazil; 2 Department of Medicine, Federal University of Rondônia, Rondônia, Brazil; 3 Institute of Anatomical Pathology, Rede D’Or São Luiz Hospitals Network, São Paulo, Brazil; 4 Department of Medicine, Centro Universitário São Camilo, São Paulo, Brazil; 5 Molecular Oncology Research Center, Barretos Cancer Hospital, Barretos, São Paulo, Brazil; 6 Life and Health Sciences Research Institute, Medical School, University of Minho, Braga, Portugal; 7 Department of Pathology, Barretos Cancer Hospital, Barretos, Brazil; National Health Research Institutes, TAIWAN

## Abstract

**Background:**

Oropharyngeal cancer is an important public health problem. The aim of our study was to correlatep16 immunohistochemistry in oropharynx squamous cell carcinomas(OPSCC) with clinical and epidemiological features.

**Material and methods:**

We conducted across-sectional study on patients with OPSCC treated at a single institution from 2014 to 2019. Epidemiological and clinical-pathological data were collected from medical records and a questionnaire was applied to determine alcohol consumption, smoking, and sexual behavior. The HPV status was determined by p16 immunohistochemistry.

**Results:**

A total of 252 patients participated in the study, of these 221 (87.7%) were male. There were 81 (32.14%) p16 positive cases and 171 (67.85%) p16 negative cases. The p16positive group was significantly associated with younger patients (50–59 years), higher education level, lower clinical stage and patients who never drank or smoked. Through univariate logistic regression, we observed that female sex (OR, 3.47; 95% CI, 1.60–7.51) and higher education level (OR, 9.39; 95% CI, 2, 81–31,38) were significantly more likely to be p16 positive. Early clinical stage (AJCC8ed) was more associated with p16 positivity both in univariate (OR, 0.14; 95% CI, 0.07–0.26, p<0.001) and multivariate analysis (OR, 0.18; 95% CI, 0.06–0.49, p = 0.001).

**Conclusion:**

This study showed that drinkers and current smokers were less likely to be p16+. Female sex, higher education level and younger age at diagnosis were associated with a higher probability of being p16+. Additionally, there was a higher proportion of patients with early clinical stage (I or II) in the p16 positive group when compared to the p16 negative group.

## Introduction

Oropharyngeal cancer is an important public health problem. According to GLOBOCAN 2018 [1], it represents 0.5% (92,887) of the total number of new cancer cases and 0.5% (51,005) of the total number of cancer deaths.

Drinking and smoking are considered serious risk factors in oropharyngeal carcinogenesis [2–4]. However, in recent decades, another important etiological factor for oropharynx squamous cell carcinomas (OPSCC) has been discovered: human papillomavirus (HPV). The prevalence of HPV in oropharyngeal tumours varies according to the year of study, population studied and method of analysis. In recent decades, HPV prevalence in these tumours has been increasing globally and are found to be more prevalent in developed countries than in developing ones [5, 6].

HPV status can be determined by several methods but, clinically, immunohistochemistry for the p16 protein has shown several benefits as it is practical, simple and inexpensive [7, 8]. Several important international guidelines, such as the National Comprehensive Cancer Network [9] and the guidelines of the American College of Pathologists [10], recommend the clinical use of p16 by immunohistochemistry to characterize cases such as HPV^+^ or HPV^−^. Additionally, the staging of oropharyngeal tumours changed in the 8th edition of the AJCC [11, 12].

The aim of our study was to correlate the p16 immunohistochemistry in OPSCC with clinical and epidemiological features.

## Material and methods

We conducted a cross-sectional study of 252 patients diagnosed with OPSCC, treated at a single tertiary referral institution for cancer treatment in Brazil from 2014 to 2019. Demographic data (sex, age, marital status) and clinical-pathological data (TNM clinical stage according to the eighth edition of the American Joint Committee on Cancer (AJCC) TNM staging system) were obtained from medical records. Of the 252 patients in the study, 125 answered a questionnaire on sexual behavior, smoking and alcohol consumption habits.

**Fig 1:**
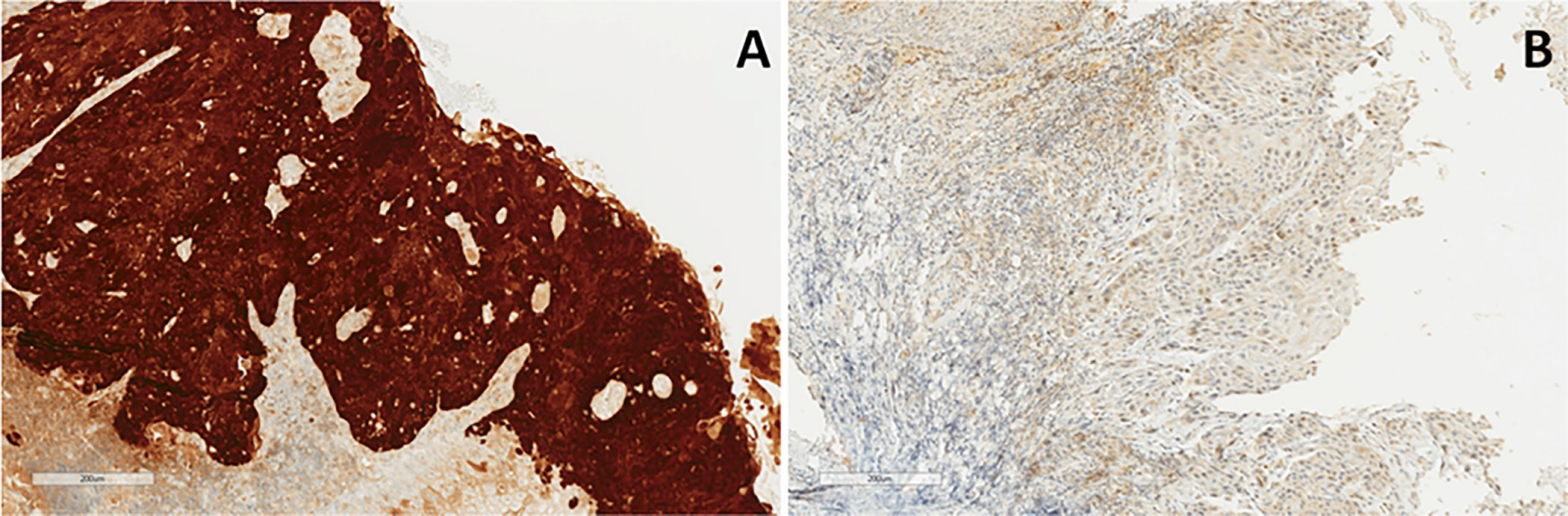
Representative photomicrograph of p16 expression (immunohistochemistry). 10x magnification. A: Oropharynx squamous cell carcinomawith p16-positive immunohistochemistry. B: Oropharynx squamous cell carcinomawith p16-negative immunohistochemistry.

HPV status was determined in all patients by p16 immunohistochemistry, which is a well-established surrogate marker to characterize HPV^+^ oropharyngeal tumours [10]. Briefly, the paraffin blocks from the biopsy or surgery of the patients were separated, and the most representative areas of these blocks containing the tumours were selected and sliced into 4-μm sections. Immunohistochemistry was performed using the CINtec® p16 Histology kit (Roche MTM Laboratories, Heidelberg, Germany), according to the manufacturer’s instructions. The expression of p16 was classified as positive in the presence of strong and diffuse staining in more than 75% of both nuclei and cytoplasms. Any other colour pattern was classified as negative (Fig 1) [13–15]. Thus, patients with p16 positivity by immunohistochemistry were considered to be HPV^+^ [16, 17].

### Ethical approval

The study was approved by the Medical Ethics Committee of the Barretos Cancer Hospital under number 1,943,689. Written informed consent was obtained from all patients who were enrolled in this study.

### Statistical analysis

The analyses were performed using the software SPSS version 21 (SPSS, Inc., Chicago, Illinois). The sociodemographic and clinical characteristics based on HPV status were analysed using the chi-squared test (or Fisher’s exact test) when the variables were qualitative and the Mann-Whitney test for quantitative variables. A p-value < 0.05 was considered statistically significant. To verify the relationships between the studied variables and HPV positivity, multivariate logistic regression was used to estimate the odds ratio (OR) and its respective 95%.

## Results

In the period from 2014 to 2019, we included a total of 252 patients diagnosed with OPSCCtreated at our institution. The sociodemographic and clinical pathological characteristics are summarised in Table 1. The majority were male (221 = 87.69%), and the most frequent age group was 50–59 years, with 85 (33.7%) cases.

**Table 1:** Associations of p16 expression with the epidemiological characteristics of the patients.

Variables	P16				P-value
	Negative		Positive		
	n	(%)	n	(%)	
**Sex**^**a**^					
Male	158	92.4%	63	77.8%	0.01*
Female	13	7.6%	18	22.2%	
**Age** ^**b**^					
20–29	1	6.1%	0	0.0%	0.005*
30–39	1	6.1%	3	3.7%	
40–49	27	15.8%	25	30.9%	
50–59	54	31.6%	31	38.3%	
60–69	57	33.3%	15	18.5%	
70–79	22	12.9%	6	7.4%	
80–89	8	4.7%	1	1.2%	
90–99	1	0.6%	0	0.0%	
**Skin colour**^**a**^					
White	92	55.4	50	64.9	0.16
Non-white	74	44.6	27	35.1	
**Schooling**^**a**^					
Illiterate	23	15.2%	7	8.6%	0.001*
Elementary school	99	65.6%	35	43.2%	
High school	22	14.6%	19	23.5%	
Higher	7	4.6%	20	24.7%	
**T**^**a**^					
T1/T2	53	31.5%	33	42.3%	0.100
T3/T4	115	68.5%	45	57.7%	
**N**^**a**^					
N0	41	24.4%	17	21.8%	0.654
N positive	127	75.6%	61	78.2%	
**M**^**b**^					
M0	161	95.8%	72	92.3%	0.357
M1	7	4.2%	6	7.7%	
**TNM staging** ^**a**^					
Early-stage I and II	22	13.1	41	52.6%	0.001*
Advanced III and IV	146	86.9%	37	47.4%	

^ª^ Analysis by the chi-squared test expressed as absolute (n) and relative (%) frequency

^b^ Analysis by Fisher’s exact test expressed as absolute (n) and relative (%) frequency

* Statistically significant difference P ≤ 0.05

Data analysis showed that 30 (12.93%) patients were illiterate, 134 (57.75%) completed elementary school, 41 (17.67%) completed high school, and 27 (11, 63%) completed higher education. The results for skin colour showed that 142 (58.43%) of the patients self-reported as white while 101 (41.56%) patients self-reported as non-white (brown, black, Asian, indigenous). The findings on marital status showed that 18.79% (133/233) of the patients had a fixed partner (married or in a stable union), 21.46% (50/233) had no fixed partner (widowed or separated), and 21.46% (50/233) were single.

A total of 81 (32.1%) patients tested positive for p16 staining while 171 (67.85%) tested negative.

Although a lower number of women participated in the study, it should be highlighted that the probability of oropharyngeal cancer in women being HPV induced based on the p16 status is higher than when a man is diagnosed with oropharyngeal cancer.

In the p16^+^group, the patients were younger, with a predominant age range of 50–59 years compared to a predominance of 60–69 years in the other group (P = 0.005). No significant differences regarding ethnicity were observed. The p16 positive patients showed significant higher education level (24.7% vs. 4.6%, P < 0.001), and a higher proportion of patients who finished high school (23.5% vs. 14.6%, P < 0.001). In the p16 negative group, a greater proportion of individuals were illiterate (15.2% vs. 8.6%, P < 0.001).

No significant differences in relation to T, N, or M stage were identified. However, in the p16negativegroup, there was a higher number of patients with advanced clinical stage (III or IV) (86.9% vs. 47.4%) and a greater proportion of patients in the p16 positive group with early clinical stage (I or II) (13.1 vs. 52.6, P <0.001) (Table 1).

Analysis of the questionnaires on patient habits showed a much higher prevalence of patients who never drank in the p16 positive group than in the p16 negative group (16% vs. 2.7%, P = 0.015). Similarly, the number of patients who never smoked in the p16 positive group was more predominant compared to the p16 negative group (32% vs. 6.7%, P < 0.001) (Table 2).

**Table 2:** Data from the questionnaire related to smoking, alcohol consumption and sexual behavior.

Variables	p16				P-value
	Negative		Positive		
	n	(%)	n	(%)	
**Alcohol**^b^					
Never drank	2	2.7%	8	16.0%	0.015*
Ex-drinker	46	61.3%	22	44.0%	
Current drinker	27	36.0%	20	40.0%%	
**Smoking** ^**a**^					
Never smoked	5	6.7%	16	32.0%	0.001*
Ex-smoker	34	45.3%	18	36.0%	
Current smoker	36	48.0%	16	32.0%	
**Number of sexual partners throughout life**^a^					
None	0	0.0%	0	0.00%	0.362
1 to 3	12	16.0%	10	20.0%	
4 to 10	27	36.0%	12	24.0%	
>10	36	48.0%	28	56.0%	
**Number of partners with whom the patient practised active oral sex throughout life**^b^					
None	37	50.7%	15	30.0%	0.126
1 to 3	27	37.0%	24	48.0%	
4 to 10	4	5.5%	5	10.0%	
>10	5	6.8	6	12.0%	
**Number of partners with whom the patient practised passive oral sex throughout life**					
None	29	39.2%	9	18.0%	0.55
1 to 3	29	39.2%	22	44.0%	
4 to 10	8	10.8	11	22.0%	
>10	8	10.8	8	16.0%	

^ª^ Analysis by the chi-squared test expressed as absolute (n) and relative (%) frequency

^b^ Analysis by Fisher’s exact test expressed as absolute (n) and relative (%) frequency

* Statistically significant difference

From the data, no significant differences were observed in the number of sexual partners throughout life, nor in the number of partners with whom the patients practised either passive or active oral sex (Table 2).

Through univariate logistic regression, we determined that female sex (OR, 3.47; 95% CI, 1.60–7.51) and higher education level (OR, 9.39; 95% CI, 2, 81–31,38) were significantly related to a higher probability of being p16 positive. Current alcohol drinking (OR, 0.14; 95% CI, 0.04–0.44) and current smoking (OR, 0.14; 95% CI, 0.04–0.44) were less associated with p16 positivity in univariate analysis. (Table 3). Early clinical stage was more associated with p16 positivity both in univariate (OR, 0.14; 95% CI, 0.07–0.26, p<0.001) and in multivariate analysis (OR, 0.18; 95% CI, 0.06–0.49, p = 0.001).

**Table 3:** Model of the univariate and multivariate analysis of sociodemographic data based on p16 status.

	Univariate				Multivariate			
Variables	Odds ratio	Confidence Interval for Odds Ratio		p-value	Odds ratio	Confidence Interval for Odds Ratio		p-value
		Lower	Higher			Lower	Higher	
**Sex**								
Male	1	-	-		1	-	-	
Female	3.473	1.606	7.506	0.002	3.872	0.808	18.558	0.09
**Alcohol**								
Never drank	1	-	-	0.032	1	-	-	0.227
Ex-drinker	0.120	0.023	0.611	0.011	0.297	0.037	2.356	0.25
Current drinker	0.186	0.186	0.035	0.046	0.593	0.065	5.372	0.642
**Smoking**								
Never smoked	1	-	-	0.003	1	-	-	0.246
Ex-smoker	0.165	0.052	0.525	0.002	0.283	0.064	1.242	0.094
Current smoker	0.139	0.043	0.445	0.001	0.354	0.077	1.62	0.181
**Education**								
Illiterate	1	-	-	< 0,001	1	-	-	0.295
elementary school	1.182	0.458	2.943	0.752	1.537	0.451	5.234	0.492
high school	2.838	0.998	8.071	0.051	2.984	0.652	13.665	0.159
higher	9.388	2.808	31.385	< 0,001	4.3	0.716	25.837	0.111
**CS**								
Early-stage	1	-	-	-	1	-	-	
Advanced	0.136	0.072	0.256	< 0,001	0.182	0.067	0.492	0.001

Reference: p16+

## Discussion

In the present study, we found that p16 positive oropharyngeal squamous-cell carcinoma was more common in patients who never drank, never smoked, in those with a higher educational level and was associated with tumours presenting in early clinical stage (I and II).

The majority of studies relating HPV to OPSCC do not follow standardised methods and, consequently, several authors use different diagnostic methods for HPV (p16, PCR), resulting in varied prevalence rates. In addition, the prevalence depends on the population studied and the year of the study. In this research, we considered p16 immunohistochemistry as a marker for HPV status in all patients.

One of the differentials of our study is the application of a questionnaire on sexual behavior and consumption of alcohol and tobacco. Although a difference between p16 positive and p16 negative patients regarding sexual behavior was not detected, some studies demonstrate the opposite.D’Souza et al. [18] determined that a history of 6 or more oral sex partners was associated with a greater risk of OPSCC, D’Souza et al. found that a number of vaginal sex partners (≥26 compared to 0–5) was associated with a greater risk of OPSCC among men and the studies of Baumeister et al. [19] and Dahlstrom et al. [20] found the same result. Our findings are corroborated by other authors: Talamini et al. [21], Garrote et al. [22] and Smith et al. [23] who did not find a significant association between lifetime number of sexual partners and the risk of oral cancer.

Our study showed a 32.14% prevalence of p16 positivity in oropharyngeal carcinomas. Most studies involving populations from developing countries identified a low incidence of HPV in oropharyngeal tumours [24, 25]. López et al. [26] reported an incidence of 6.6% in Brazil and Anantharaman et al. [6] compared cases from the USA, Europe, and Brazil and found an incidence of only 4.1% of positivity in the population studied in Brazil. Petito et al. [16] studied 82 cases in Brazil and found an incidence of 25.6%, Betiol et al. [27] found 17.7% and de Cicco et al. [28] reported 59.1%. A more recent study in a developing country was that of Bahl et al. [29] who found a 22.8% incidence of HPV in oropharyngeal tumours in India. In developed countries, Nasman et al. [30] studied 98 patients with oropharyngeal cancer in Sweden and found an HPV incidence of 79%.

Most of the patients in our study, in both p16^+^ and p16^−^ groups, had advanced clinical stage (III or IV). However, specifically in the p16^+^ group, there was a proportionally larger number of patients with early clinical stages (I and II) compared to the p16^−^ group (52.6% vs. 13.1%, P < 0.001). De Cicco et al. [28] and Du et al. [31] reported similar results.

Based on the biological behavior of induced HPV oropharyngeal cancer, there was a change in staging in the eighth edition, generating its own staging for the positive p16. This made it possible to perform a downstage of this population in comparison with the negative p16. Therefore, what is seen in the results are tumours that are now considered to be initial due to the new staging, which is inherent in the staging, which better reflects the positive p16 neoplasia and its consequent better prognosis.

According to Vokes et al. [32] this difference in prognosis by p16 immunohistochemical status was considered in the change in staging that occurred between the 7th and 8th edition of the TNM. In the seventh, only T1 or T2 N0 and M0 were considered early-stagetumours, regardless of their HPV status. In the eighth edition, the HPV-negative tumours continued to be considered early stage T1 or T2 N0 and M0, but among the HPV^+^ tumors, T3 and N2 were also considered early stage. Although the 8th edition of the TNM staging manual separates the staging of oropharyngeal squamous-cell carcinomas according to HPV positivity, the treatment for HPV-associated oropharyngeal carcinomas remains the same for HPV^−^tumours, except in clinical trials [32].

In Table 4, we observe the differences between the 7th and 8th edition of the TNM (Table 4) and in particular that in the positive p16 all T3 / N2 are stage II, all T4 / N3 are stage III and stage IV only for M1.

**Table 4:** Major changes from the seventh to the eighth editions of TNM [12].

Edition of TNM	Main characteristics
7Th edition	1-presence of T0 and Tis
	2-no emphasis on extra-capsular extension
	3-division of T4 into a and b
	4-stage IV for disease T4, N2, N3 and M1
	5-number of metastatic lymph nodes differed from N2a to N2b
8Th edition	1-absence of T0 in negative p16 and absence of Tis for positive p16
	2-creation of extra-capsular extension (N3b) only for 16 negatives
	3-absence of T4b for positive p16
	4-stage IV only for M1 disease in positive p16 5-without distinction of number of lymph nodes in positive p16

In both the univariate and multivariate analyses in our study, patients with advanced clinical stages (III and IV) were less likely to be p16^+^. This finding does not corroborate with that of Mehanna et al. [33]. On the other hand, no association was observed in our study between tumour size or the presence of cervical or distant metastases and positivity to p16. Our findings corroborate those found by López et al. [26]

Our findings showed a greater predominance of patients who had never smoked in the p16^+^ group than in the p16^−^group (32% vs. 6.7%, P <0.001). López et al. [26] reported a proportion of 14.3% of patients who never smoked among the HPV^+^ patients and 3.4% among the HPV^−^patients. Additionally, a higher proportion of current smokers among HPV^−^ patients was observed(69.8% vs. 57.1%). The study by Bahl et al. [29] also identified a higher proportion of patients who had never smoked in the HPV^+^ group (17% vs. 11%), but this was not statistically significant (p = 0.86). Other studies have also found a difference in the prevalence of p16 immunohistochemistry positivity with smoking status [24, 28]. In clinical practice, we observed that this difference is explained by the etiology of HPV^−^ cases, most of which are related to drinking and smoking.

Although we only analysed 125 of the 252 patients who answered the questionnaire on smoking habits, alcohol consumption, and sexual behavior, the smoking and alcohol consumption variables were statistically significant. There was a higher prevalence of patients who never drank in the p16^+^ group than in the p16^−^ group (16% vs. 2.7%, P = 0.015). De Cicco et al. [28] and López et al. [26] found similar results. In comparison, Bahl et al. [29] did not find evidence of a correlation between alcohol consumption and HPV positivity (P = 0.18).

There was a greater predominance of males in the p16^−^ group (92.4% vs. 77.8%, P = 0.01) and this finding is in agreement with those reported by López et al. [26] Petito et al. [16] the ICON-S study [34], Bahlet al. [29] and De Cicco et al. [28]. In most cases, males are more likely to have tumours of the head and neck, mainly due to their greater exposure to alcohol and smoking compared to women. The same result was found in the in the p16^−^ group.

Although most patients were male in both the HPV^+^ and HPV^-^groups, the probability of a woman being HPV^+^ was greater than being HPV^-^ negative.

The age of the patients in the study ranged from 26 to 98 years, with a mean of 60.5 years. The most frequent age group was 50–59 years, with 85 (33.73%) patients. In the p16^+^ group, the patients were younger, with a predominant age range of 50–59 years vs. 60–69 years in the p16^−^group (P = 0.005). The data of our study is in agreement with the worldwide literature which shows that HPV^+^ tumours affect younger patients [16, 29]. In contrast, de Cicco did not find significant differences in age between HPV^+^ and HPV^−^ patients [28].

As regards education level, the group with p16^+^ immunohistochemical examination had more patients with higher education(24.7% vs. 4.6%, P < 0.001) and patients with a high-school education (23.5% vs. 14.6%, P < 0.001). In the p16^−^ group, there was a greater proportion of individuals who were illiterate or who could only read and write (15.2% vs. 8.6%, P < 0.001). Our results corroborate worldwide literature which correlates HPV^+^ oropharyngeal tumours with patients with higher educational levels [35, 36].

The following variables were not statistically significant in our study: number of sexual partners throughout life and number of partners with whom the patients practiced either passive or active oral sex. These findings are not in accordance with the current literature, where sexual behavior is correlated with HPV positivity [10, 29, 37–41]. It is possible that our patients were more reticent about answering questions on sexual behavior due to embarrassment or shyness.

In our study we found that drinkers and current smokers had a lower chance of having a p16^+^ immunohistochemical test. Female sex and higher education level were associated with a higher probability of a p16^+^ immunohistochemical test. We did not find any correlation between the number of sexual partners throughout life or number of partners with whom the patient practiced oral sex (passive or active) throughout life and p16^+^ immunohistochemical test. These results are relevant because they demonstrate that p16-positive oropharyngeal tumours have a different epidemiological profile. Based on its biological behavior and clinical presentation, HPV^+^ oropharyngeal tumour is a pathology that requires further study and broader understanding in order to better optimize and stratify the care and treatment of patients with oropharyngeal cancer.

## Supporting information

S1 File (XLSX)
